# A longitudinal study to investigate previous *Chlamydia trachomatis* infection as a risk factor for subsequent anorectal infection in men who have sex with men (MSM) and women visiting STI clinics in the Netherlands

**DOI:** 10.1017/S0950268819001018

**Published:** 2019-06-13

**Authors:** J. Leenen, G.A.F.S. van Liere, C.J.P.A. Hoebe, A.A. Hogewoning, H.J.C. de Vries, N.H.T.M. Dukers-Muijrers

**Affiliations:** 1 Department of Sexual Health, Infectious Diseases and Environmental Health, South Limburg Public Health Service (GGD Zuid Limburg), Heerlen, the Netherlands; 2 Department of Medical Microbiology, Care and Public Health Research Institute (CAPHRI), Maastricht University Medical Center (MUMC+), Maastricht, the Netherlands; 3 STI Outpatient Clinic, Regional Public Health Service of Amsterdam (GGD Amsterdam), Amsterdam, the Netherlands; 4 Department of Infectious Diseases, Regional Public Health Service of Amsterdam (GGD Amsterdam), Amsterdam, the Netherlands; 5 Amsterdam Infection and Immunity Institute (AI&II), Academic Medical Center (AMC), University of Amsterdam, Amsterdam, the Netherlands; 6 Department of Dermatology, Academic Medical Center (AMC), University of Amsterdam, Amsterdam, the Netherlands

**Keywords:** Anorectal, *Chlamydia trachomatis*, gastrointestinal tract, oropharyngeal

## Abstract

Although anorectal *Chlamydia trachomatis* (CT) infections are frequently diagnosed in men who have sex with men (MSM) and women, the reason for this infection often remains unexplained, as anal sex is not always reported. Oropharyngeal infections inoculating the gastrointestinal (GI) tract may contribute to anorectal-CT infections, as evidence in animals suggests that chlamydia bacteria undergo GI passage; however, no evidence exists in humans. Longitudinal patient clinic-registry data from MSM (*n* = 17 125) and women (*n* = 4120) from two Dutch sexually transmitted infection clinics were analysed. When adjusting for confounding socio-demographics, co-infections and risk behaviour, previous (from 3 weeks up to 24 months) oropharyngeal CT was not a risk factor for subsequent anorectal CT in women (odds ratio (OR) 0.46; 95% confidence interval (CI) 0.18–1.18; *P* = 0.11) and MSM (OR 1.33; 95% CI 0.86–2.07; *P* = 0.204). Despite the large dataset, the numbers did not allow for the estimation of risk in specific subgroups of interest. The role of the GI tract cannot be excluded with this epidemiological study, but the impact of preceding oropharyngeal CT on anorectal-CT infection is likely limited.

## Introduction

Anorectal infections with *Chlamydia trachomatis* (CT) are commonly found in men who have sex with men (MSM) and in women. The prevalence is reported to be between 1% and 18% for MSM and between 6% and 17% for women [1–5]. Anorectal-CT infections are often asymptomatic (36–100%) [1, 6, 7] and are frequently (~50–65%) detected in MSM and women who did not report anal sex [6, 8, 9]. In women, self-infection (autoinoculation) from the genital site is postulated as an explanation for the detection of anorectal CT in the absence of anal sex, as most anorectal infections co-occur with genital-CT infections in women [10]. Another theory to explain anorectal-CT infections in women and MSM involves the oropharyngeal site and the gastrointestinal (GI) tract serving as a reservoir for CT [11–13]. There is an on-going debate on whether humans can host CT bacteria in the intestine and develop an anorectal infection via contamination from the lower GI tract, as described in several animal studies [11, 12, 14].

*Chlamydia* species are commensal bacteria in the gut of many animals, such as mice, birds, sheep and cattle [11]. Chlamydia bacteria have been proven to be able to survive in the GI tract of animals for a long time (up to 3 years) without causing an immune reaction through down-regulation of the immune system in the gut, as no inflammatory response was seen in histopathological examination of chlamydia-infected tissue [11, 15, 16]. In a study by Yeruva *et al*., mice were orally infected with *Chlamydia muridarum*; after 10 days, the bacteria could be detected in the caecum and large intestine. Chlamydia might survive the acidic environment of the stomach and remain in the lower intestinal tract [12]. Studies have shown that animals with chlamydia bacteria in the GI tract continue to shed organisms for a long period of time, even up to 4 years [12, 16]. Chlamydia bacteria seem to be able to pass through the GI tract to the anorectal area, at least in animals. Although some evidence for an oropharyngeal–anorectal route exists in animals, no conclusive relationship between oropharyngeal CT (or lymphogranuloma venereum (LGV)) and the GI tract in humans has been demonstrated yet.

The only evidence in humans includes studies with infants born to CT-infected mothers. Those studies conclude that, persistent GI chlamydial infection might also occur in humans [14, 17]. In a study by Schachter *et al*., some of the infants born to CT-infected mothers became CT-colonised in the anorectal region after 41–79 days of age. This later onset raised questions regarding if CT colonisation in the GI tract was possible in these infants [14]. Nevertheless, it is debatable whether positive anorectal cultures are the result of chlamydia bacteria in the intestine. Infants could also become CT-infected via the respiratory tract, rectum or vagina through perinatal exposure [14].

Bavoil *et al*. hypothesised that active oral sex (fellatio) could lead to the colonisation of the GI tract with infectious chlamydia bacteria and, from there, contaminate and infect the rectum and female genital tract [13]. For LGV, a specific CT type that mainly occurs in MSM, it was carefully suggested that oropharyngeal infections might play a role in inducing anorectal LGV via the GI tract, thereby potentially contributing to its on-going transmission [18]. However, the relationship between oropharyngeal and anorectal-CT infection in humans has not yet been studied extensively, because human experiments are hampered for medical ethical reasons, and therefore, whether such association exists remains unclear.

Another approach to study this is through the use of retrospective clinical data of patients who visited a sexually transmitted infection (STI) clinic multiple times. In the current study, we analysed the association between preceding oropharyngeal and subsequent anorectal CT in MSM and women using a large set of retrospective patient clinic-registry data from two STI clinics.

## Methods

**Fig. 1.**
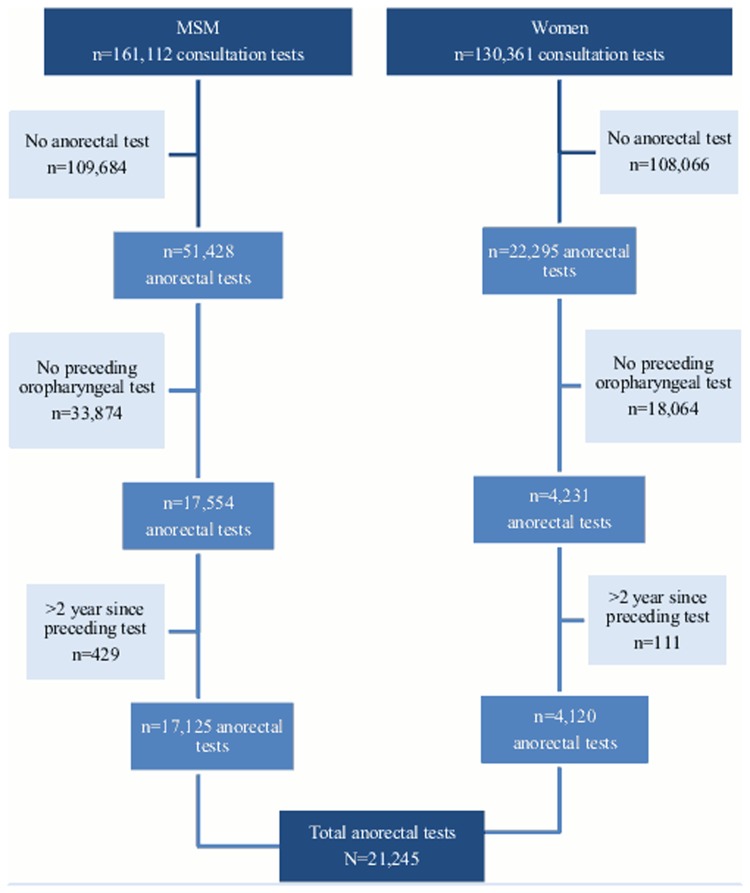
Flow chart of study population between January 2006 and December 2013 (testing consultations).

The outpatient STI clinics of the Public Health Services in Amsterdam and South Limburg offer free STI testing to at-risk groups with and without symptoms, including those attending after partner notification. Women and MSM aged 16 years and older, who visited the STI clinic from January 2006 to December 2013, were included (see Fig. 1).

Because the retrospective coded data originated from standard care and were analysed anonymously, neither a full ethical review nor informed consent for data analysis was needed, as confirmed and approved by the Medical Ethical Committee of Maastricht University (METC 11-4-108).

### Study procedures: testing

Patients were routinely tested for urogenital CT and *Neisseria gonorrhoeae* (NG). MSM were tested for anorectal and oropharyngeal CT when indicated (i.e. after the self-report of anal sex or symptoms). From 2010 onwards, MSM were routinely tested for anorectal and oropharyngeal CT in South Limburg. In Amsterdam, MSM were routinely tested for oropharyngeal CT since 2011. Women who were notified, reported symptoms or were paid for sex were tested for anorectal and oropharyngeal CT (since 2011, Amsterdam) [19].

Specimens tested for CT consisted of urine (MSM), self-collected vaginal and/or anorectal swabs, nurse-collected oropharyngeal swabs and clinician-collected cervical and urethral swabs. Tests were performed according to the manufacturer's protocol. In South Limburg, specimens were processed at two regional laboratories using three different nucleic acid amplification assays (SDA, Becton Dickinson ProbeTec ET system, Maryland, USA, until 2012; Cobas Amplicor, Roche, California, USA, 2006–2011; Cobas 4800, Roche, California, USA, since 2012). In Amsterdam, from 2008 the Aptima Combo 2 Assay for CT/NG has been used to detect rRNA (Hologic Gen-Probe Inc., San Diego, USA). Before 2008, the Cobas Amplicor was used. Culture was also used in case of symptoms, being notified for STI, paid for sex or MSM for oropharyngeal (until 2008), urogenital and anorectal NG. Each consultation included a standardised nurse-collected medical and sexual history [19].

## Statistical analyses

### Dataset

Two subsequent visits from one person were taken as a measurement pair, based on an anonymised person identifier. Between the first (preceding) and second (subsequent) visit (measurement pair), there was a time-window ranging from 21 days up to 730 days. This timeframe was chosen because the bacteria may need time to reach the GI tract, and it has been found that animals with chlamydia bacteria in their gut continued to shed chlamydia organisms for several years [12, 16].

A person could be included in the dataset with multiple measurement pairs (or so-called repeated measurements) when he or she visited the STI clinic more than two times between 2006 and 2013. Measurement pairs were included in analyses when the preceding visit had (at least) an oropharyngeal-CT test, and the subsequent visit had (at least) an anorectal-CT test.

Missing values were treated as a separate category, except for where the number of missing values was small (<25). In such case missing values were attributed to the most likely value, i.e. cases that had missing results for preceding urogenital CT (*n* = 6), subsequent urogenital CT (*n* = 10), urogenital NG (*n* = 21) and anorectal NG (*n* = 18) were defined as negative.

### Generalised estimating equation (GEE)

Univariate and multivariate GEE analyses were used to estimate the association between preceding (⩽24 months) oropharyngeal CT and subsequent anorectal CT. GEE analysis took into account the repeated measurements and therefore corrected for individuals bringing more measurement pairs into the data than others.

All analyses were stratified for MSM and women because of the different testing guidelines for both groups [1]. For MSM, all univariate confounders were statistically significant (*P* < 0.05) and thus added to the multivariate model. For women, a multivariable model was constructed by adding variables in groups (socio-demographic, co-infections and risk behaviour) to the model using a stepwise backward approach, and thus not included variables that were not statistically significant in univariate analyses.

### Main effect

To assess the association between preceding oropharyngeal and subsequent anorectal CT, oropharyngeal CT at the preceding consultation was defined as the main exposure variable of interest. A priori, as epidemiological associations may be subject to confounding, the main effect was adjusted for several confounding factors. Correction was deemed essential, especially in studying this association, as oropharyngeal-CT infection may also represent high risk sexual behaviour, which is also highly associated with anorectal CT [1, 4].

### Confounders

General socio-demographic confounders included in the model were age (<30, 30–45, >45 years) and STI clinic (Amsterdam, South Limburg).

Co-infections with oropharyngeal CT, urogenital CT, urogenital NG, anorectal NG, HIV (MSM) and syphilis, as well as preceding anorectal- and urogenital-CT and -NG infections were considered important surrogate markers for risk behaviour (i.e. unsafe sex) and considered as potential confounders.

Other proxies for risk behaviour included being notified for STI, new sex partners in the past 6 months (data only available from the STI clinic in Amsterdam), number of sex partners in the past 6 months and self-reported receptive anal sex in the past 6 months. Genital and anorectal symptoms were testing indications and therefore included in the model. In MSM, receptive anal sex is defined as such when (1) MSM were tested anorectally before 2012 at the STI clinic South Limburg and Amsterdam, (2) receptive anal sex was reported at the STI clinic in Amsterdam since 2012 or (3) anal sex was reported at the STI clinic in South Limburg for the whole study period. Guidelines advised to treat chlamydia infections with a single dose azithromycin 1000 mg [20]. From 2012, national guidelines advised to treat anorectal chlamydia infections with doxycycline 100 mg two times per day for 7 days [21]. In Amsterdam, patients were treated with doxycycline for anorectal chlamydia infections during the whole study period (personal communication).

We considered a *P*-value of <0.05 as statistically significant in univariate and multivariate analyses. All analyses were performed using SPSS version 20.0 (IBM Inc., Somers, NY, USA).

### Sensitivity analyses

Since testing guidelines changed after 2010, additional analyses were performed by restricting data to tests from 2010 onwards. Other sensitivity analyses were performed by comparing women who reported anal sex with those who did not report anal sex. Due to low subgroup numbers (*n* = 5), risk factor analyses for MSM who did not report anal sex or for patients who had a single-anorectal infection was not possible due to the low numbers of preceding oropharyngeal infections in the included measurement pairs of these sensitivity analyses (Table 1).

**Table 1.** Subgroups with oropharyngeal-CT infection on the preceding visit

	*N*	Subsequent anorectal positivity *n* (%)
*Timeframe*		
MSM		
<6 months	139	17 (12.2%)
<12 months	191	25 (13.1%)
Women		
<6 months	60	8 (13.3%)
<12 months	74	8 (10.8%)
*Concurrent urogenital CT*		
MSM		
Positive	1	1 (100%)
Negative	7	4 (57.1%)
Not tested	200	25 (12.5%)
Women		
Positive	10	9 (90%)
Negative	70	0 (0%)

Descriptive sensitivity analyses included restrictions to shorter time intervals between measurement pairs (⩽12 months and ⩽6 months) and with patients who had a single-anorectal CT (without concurrent urogenital CT). This was done to obtain insight into the effect of concurrent urogenital infections (see Table 1).

## Results

### Characteristics

The analyses included 21 245 measurement pairs consisting of a preceding clinic visit with (at least) an oropharyngeal-CT test and a subsequent clinic visit with an anorectal-CT test. MSM contributed 17 125 (80.6%) measurement pairs, and women contributed 4120 (19.4%) measurement pairs. Every individual had at least one measurement pair, with a maximum of 24 pairs. The data included 7272 unique individuals: 5493 MSM (75.5%) and 1779 women (24.5%). For MSM, the median age was 40 (range: 16–79, interquartile range (IQR): 33–47) in Amsterdam and 43 (range: 16–74, IQR: 32–50) in South Limburg (*P* < 0.001). For women, the median age was 27 (range: 17–66, IQR: 23–34) in Amsterdam and 44 (range: 18–63, IQR: 38–49) in South Limburg. At the first (preceding) visit of the measurement pairs, oropharyngeal-CT positivity was 1.2% for MSM (*n* = 208) and 1.9% for women (*n* = 80). At the subsequent visit of the measurement pairs, anorectal-CT positivity was 7.7% for MSM (*n* = 1316) and 5.4% for women (*n* = 224).

### Main effect unadjusted

In univariate analyses, preceding oropharyngeal CT was associated with subsequent anorectal CT in MSM (odds ratio (OR) 2.05, 95% confidence interval (CI) 1.40–3.01, *P* < 0.0001) and in women (OR 2.26, 95% CI 1.06–4.80, *P* = 0.04) (Tables 2 and 3).

**Table 2.** Absolute numbers and prevalences of anorectal CT infections and univariate and multivariate risk factors using GEE analyses in MSM

	Characteristics		Anorectal positive		Univariate^a^	Multivariate ^a,b^
Main effect	*n*	(%)	*n*	(%)	OR (95% CI)	OR (95% CI)
Previous oropharyngeal CT						
Positive	208	1.2	30	14.4	2.05 (1.40, 3.01)***	1.33 (0.86, 2.07)
Negative	16 917	98.8	1286	7.6	1	1
*Confounding*						
Socio-demographic						
Age (years)						
<30	2970	17.3	261	8.8	1.46 (1.21, 1,76)***	1.52 (1.25, 1.85)***
30–45	8409	49.1	699	8.3	1.37 (1.18, 1.60)***	1.31 (1.13, 1.52)***
>45	5746	33.6	356	6.2	1	1
STI clinic location						
Amsterdam	13 241	77.3	1108	8.4	1.61 (1.33, 1.95)***	1.26 (0.94, 1.69)
Limburg	3.884	22.7	208	5.4	1	1
Co-infections						
Current oropharyngeal CT						
Not tested	68	0.4	6	8.8	1.25 (0.54, 2.90)	1.48 (0.57, 3.84)
Positive	187	1.1	99	52.9	14.56 (10.76, 19.70)***	11.82 (8.26, 16.89)***
Negative	16 870	98.5	1211	7.2	1	1
Current urogenital CT						
Not tested	59	0.3	5	8.5	1.29 (0.52, 3.24)	1.16 (0.43, 3.13)
Positive	586	3.4	210	35.8	7.81 (6.49, 9.39)***	6.12 (4.94, 7.57)***
Negative	16 480	96.2	1101	6.7	1	1
Previous urogenital CT						
Not tested	56	0.3	5	8.9	1.20 (0.48, 3.03)	1.11 (0.40, 3.10)
Positive	637	3.7	71	11.1	1.52 (1.17, 1.95)**	0.90 (0.67, 1.20)
Negative	16 432	96.0	1240	7.5	1	1
Previous anorectal CT						
Not tested	1082	6.3	81	7.5	1.07 (0.84, 1.36)	1.28 (0.98, 1.66)
Positive	1422	8.3	207	14.6	2.25 (1.89, 2.67)***	1.53 (1.26, 1.86)***
Negative	14 621	85.4	1028	7.0	1	1
Current genital NG						
Not tested	43	0.3	2	4.7	0.60 (0.15, 2.42)	0.70 (0.15, 3.13)
Positive	361	2.1	57	15.8	2.31 (1.75, 3.05)***	0.77 (0.54, 1.08)
Negative	16 721	97.6	1257	7.5	1	1
Current anorectal NG						
Not tested	48	0.3	4	8.3	1.26 (0.45, 3.52)	1.01 (0.32, 3.15)
Positive	877	5.1	218	24.9	4.58 (3.85, 5.44)***	2.91 (2.39, 3.56)***
Negative	16 200	94.6	1094	6.8	1	1
Current HIV						
Not tested	48	0.3	6	12.5	2.54 (1.17, 5.56)*	1.88 (0.77, 4.61)
Positive	6072	35.5	725	11.9	2.41 (2.13, 2.74)***	2.06 (1.79, 2.38)***
Negative	11 005	64.3	585	5.6	1	1
Current Syphilis						
Not tested	1531	8.9	95	6.2	0.80 (0.62, 1.03)	1.25 (0.91, 1.71)
Positive	387	2.3	58	15.0	2.13 (1.60, 2.84)***	1.26 (0.92, 1.74)
Negative	15 207	88.8	1163	7.6	1	1
Risk behaviour						
Receptive anal sex						
Unknown	2637	15.4	175	6.6	4.07 (2.81, 5.88)***	3.16 (2.16, 4.61)***
Yes	12 388	72.4	1104	8.9	5.60 (4.00, 7.84)***	2.73 (1.91, 3.90)***
No	2096	12.2	36	1.7	1	1
Self-reported symptoms						
Yes	3787	22.1	495	13.1	2.29 (2.02, 2.59)***	1.43 (1.25, 1.64)***
No	13 338	77.9	821	6.2	1	1
Being notified for STI						
Yes	2531	14.8	370	14.6	2.48 (2.17, 2.83)***	1.76 (1.52, 2.04)***
No	14 594	85.2	946	6.5	1	1
New sex partner						
Yes	13 773	80.4	1143	8.3	1.66 (1.40, 1.97)***	1.02 (0.78, 1.33)
No	3352	19.6	173	5.2	1	1
Number of sex partners in the past 6 months						
Unknown	170	1.0	14	8.2	1.62 (0.93, 2.83)	1.41 (0.72, 2.75)
>3	12 128	70.8	1049	8.6	1.71 (1.48, 1.98)***	1.40 (1.12, 1.75)**
⩽3	4827	28.2	253	5.2	1	1

CT, *Chlamydia trachomatis*; NG, *Neisseria gonorrhoeae*; STI, sexually transmitted infection; MSM, men who have sex with men; CI, confidence interval.

^a^CIs that do not overlap the null value of odds ratio = 1 are shown in bold.

^b^Controlled for socio-demographic factors and proxies for risk behaviour.

*P < 0.05, **P < 0.01, ***P < 0.001.

**Table 3.** Absolute numbers and prevalences of anorectal CT infections and univariate and multivariate risk factors using GEE analyses in women

	Characteristics		Anorectal positive		Univariate^a^	Multivariate^a,b^
Main effect	*n*	(%)	*n*	(%)	OR (95% CI)	OR (95% CI)
Previous oropharyngeal CT						
Positive	80	1.9	9	11.3	2.26 (1.06, 4.80)*	0.46 (0.18, 1.18)
Negative	4040	98.1	215	5.3	1	1
*Confounding*						
Socio-demographic						
Age (years)						
<30	1566	38.0	122	7.8	2.43 (1.54, 3.83)***	2.01 (1.11, 3.62)*
30–45	1633	39.6	71	4.3	1.31 (0.80, 2.13)	1.52 (0.82, 2.79)
>45	921	22.4	31	3.4	1	
STI clinic location^c^						
Amsterdam	2321	56.3	149	6.4	1.58 (1.15, 2.16)**	
Limburg	1799	43.7	75	4.2	1	
Co-infections						
Current oropharyngeal CT						
Not tested	386	9.4	40	10.4	2.65 (1.83, 3.84)***	1.64 (0.96, 2.80)
Positive	47	1.1	30	63.8	40.49 (22.47, 72.96)***	13.28 (4.67, 37.73)***
Negative	3687	89.5	154	4.2	1	
Current urogenital CT						
Positive	237	5.8	157	66.2	111.78 (76.51, 163.28)***	95.26 (63.05, 143.95)***
Negative	3883	94.0	67	1.7	1	1
Previous urogenital CT^c^						
Positive	302	7.3	41	13.6	3.12 (2.15, 4.52)***	
Negative	3818	92.7	183	4.8	1	
Previous anorectal CT						
Not tested	1151	27.9	84	7.3	1.80 (1.35, 2.39)***	1.78 (1.17, 2.69)**
Positive	182	4.4	23	12.6	3.30 (1.99, 5.47)***	3.00 (1.36, 6.59)**
Negative	2787	67.6	117	4.2	1	
Current genital NG^c^						
Positive	59	1.4	12	20.3	4.64 (2.44, 8.82)***	
Negative	4061	98.6	212	5.2	1	
Current anorectal NG^c^						
Positive	48	1.2	11	22.9	5.39 (2.74, 10.59)***	
Negative	4072	98.8	213	5.2	1	
Risk behaviour						
Receptive anal sex^c^						
Unknown	681	16.5	26	3.8	0.92 (0.57, 1.48)	
Yes	1731	42.0	127	7.3	1.83 (1.35, 2.47)***	
No	1708	41.5	71	4.2	1	
Self-reported symptoms^c^						
Yes	633	15.4	52	8.2	1.73 (1.24, 2.41)**	
No	3487	84.6	172	4.9	1	
Being notified for STI						
Yes	272	6.6	39	14.3	3.31 (2.26, 4.87)***	1.97 (1.07, 3.63)*
No	3848	93.4	185	4.8	1	1
New sex partner^c^						
Yes	3586	87.0	179	5.0	0.57 (0.41, 0.80)**	
No	534	13.0	45	8.4	1	
Number of sex partners in the past 6 months^c^						
Unknown	113	2.7	6	5.3	0.61 (0.26, 1.43)	
>3	692	16.8	58	8.4	0.55 (0.40, 0.76)***	
⩽3	3315	80.5	160	4.8	1	

CT, *Chlamydia trachomatis*; NG, *Neisseria gonorrhoeae*; STI, sexually transmitted infection; MSM, men who have sex with men; CI, confidence interval.

^a^CIs that do not overlap the null value of odds ratio = 1 are shown in bold.

^b^Controlled for socio-demographic factors and proxies for risk behaviour.

^c^Not included in the final model by the back-step procedure.

*P < 0.05, **P < 0.01, ***P < 0.001.

### Main effect adjusted for confounders

For MSM, the addition of socio-demographic confounders to the model decreased the OR from 2.05 (95% CI 1.40–3.01, *P* < 0.0001) to 1.97 (95% CI 1.33–2.91, *P* = 0.001). By adding co-infections, the OR decreased further to 1.35 (95% CI 0.87–2.10, *P* = 0.180), and, by adding proxies for risk behaviour, the OR decreased to 1.33 (95% CI 0.86–2.07, *P* = 0.204).

For women, the addition of socio-demographic confounders to the model decreased the OR from 2.26 (95% CI 1.06–4.80, *P* = 0.04) to 1.98 (95% CI 0.93–4.21, *P* = 0.08). Adding co-infections further decreased the OR to 0.48 (95% CI 0.19–1.20, *P* = 0.12) and by adding proxies for risk behaviour to OR 0.46 (95% CI 0.18–1.18, *P* = 0.11).

### Confounders in the model and their association with anorectal CT

Associated factors for the subsequent anorectal CT in MSM were concurrent oropharyngeal CT, urogenital CT, anorectal NG, HIV, preceding anorectal CT, younger age (<34 years and between 30 and 45 years), self-reported anal sex, report of genital or anal symptoms and notification of an STI (Table 2). For women, risk factors for anorectal CT were younger age (<34 years), concurrent oropharyngeal CT, urogenital CT, preceding anorectal-CT infection and notification of an STI (Table 3).

### Sensitivity analyses for the main effect

When restricting to a shorter time interval, the OR for MSM was 1.21 (95% CI 0.77–1.92, *P* = 0.41) for ⩽12 months between preceding and subsequent visit and 1.15 (95% CI 0.68–1.93, *P* = 0.61) for ⩽6 months. For women, the adjusted OR was 0.54 (95% CI 0.21–1.39, *P* = 0.20) for ⩽12 months and the adjusted OR was 0.73 (95% CI 0.29–1.86, *P* = 0.51) for ⩽6 months.

Of the women who had a preceding oropharyngeal-CT infection, none had subsequent anorectal-only CT infection (without concurrent urogenital CT), and nine had both anorectal- and urogenital-CT infection (see Table 1).

When restricting data from 2010 onwards, the adjusted OR for MSM was 1.21 (95% CI 0.76–1.91, *P* = 0.42). For women, the adjusted OR was 0.44 (95% CI 0.16–1.17, *P* = 0.10).

The adjusted OR for women who did not reported anal sex was 0.37 (95% CI 0.08–1.7, *P* = 0.20) and for women who reported anal sex the OR was 0.39 (95% CI 0.09–1.66, *P* = 0.20).

## Discussion

Preceding oropharyngeal-CT infection is not an independent risk factor for subsequent anorectal-CT infection in MSM and women, using epidemiological methods.

Because a causal relationship between oropharyngeal and anorectal CT cannot be studied through human experiments due to medical ethical restrictions, epidemiological assessment using a retrospective longitudinal design is the next best approach. In this study, using patient clinic-registry data, a large number of MSM and women (*n* = 7272) screened for oropharyngeal CT were included in analyses. To the best of our knowledge, this is the first large human study assessing oropharyngeal CT as a predictor of subsequent anorectal CT using longitudinal data. It has been shown that many factors are epidemiologically associated with anorectal CT [22–24]. Therefore, the availability of a broad range of both socio-demographic factors and risk behaviour factors enabled adjustments for confounding factors, and the adjustment for these in analyses is a major asset of this study.

GEE analysis was considered to be the most suitable analysis for this study with repeated measurements. Analysis of variance and multivariate analysis of variance are other types of analyses for repeated measurements; however, these analyses are not able to incorporate covariates. Logistic regression analyses do not take repeated measurements into account, and survival analyses do not take into account repeated measurements from the same individual.

However, this retrospective cohort study is not without limitations. First, due to our clinics' testing policy (as in international guidelines [1]), only a select group of high-risk patients (patients who were notified, reported symptoms or commercial sex workers) were oropharyngeally tested. This could lead to an overestimation of the prevalence of oropharyngeal CT and an underestimation of the absolute number of oropharyngeal infections; however, the direction of possible bias in the risk estimates is unknown. Also, testing guidelines changed during the study period, which could lead to sampling bias of our dataset. This may influence the generalisability of our sample; it should not affect the biological association of previous oropharyngeal and subsequent anorectal CT. When restricting data to include only data collected during the latest guidelines (from 2010 onwards), sensitivity analyses showed similar results as when including data from all years.

Second, exposure may be misclassified when there were oropharyngeal infections that occurred after a clinic visit or spontaneously resolved before the visit [25], as these would be missed as exposures in the analyses. This may lead to an underestimation of the risk of anorectal CT. However, such bias may be considered limited, especially because oropharyngeal-CT infections have been found to have a low bacterial load [25] and therefore may not survive the GI tract. On the other hand, multiple low load oropharyngeal infections could accumulate in the GI tract and result in a rectal infection. However, there is no scientific evidence for survival and accumulation of CT in the human GI tract. In subcategories with missing values, where the number of cases was small, missing values were attributed to the most likely value. Although it is possible that these missing values were wrongly attributed, the numbers were small and thereby unlikely to affect the main effect.

Third, in spite of correction of confounders, we could not completely rule out residual confounding, such as confounding due to the lack of information on several variables. Because our study used routinely collected data originated from standard care, there was no information available on condom use, bacterial load, whether patients swallowed ejaculate, genotyping and the given treatment regime. Because genotyping was not available, we could not identify whether anorectal-CT infections originated from the same bacteria as the preceding oropharyngeal infections. In our study design, we hypothesise that the oropharyngeal and anorectal infection are of the same genotype. Therefore, the effect of the oropharyngeal–anorectal CT hypothesis could be overestimated, as in real life genotypes could be different. Yeruva *et al*. showed in mice that although azithromycin is able to clear the genital tract, but is unable to eliminate chlamydial infection in the GI tract with the same dose within the same animal [26]. This suggests that the GI tract may have a differential susceptibility of chlamydiae to azithromycin than the genital tract and thus could possibly reflect failure of antibiotic treatment for the GI tract. We could not assess the effect of this phenomenon on our study results, as the exact treatment regime received by patients was not recorded in the routinely collected data originating from standard care used in this study.

Fourth, although this study included a large number of measurement pairs, these numbers did not allow for the estimation of risks in specific subgroups of interest (such as MSM with a preceding oropharyngeal infection who did not report anal sex). For example, in women, genital infection may lead to anorectal positivity by autoinoculation, which can have an impact on the number of anorectal infections, thereby influencing the relationship found between oropharyngeal and anorectal infections [4, 10, 23, 27]. Although we corrected for urogenital-CT infections in the model, the number of women with anorectal-only CT infections (and in whom the role of autoinoculation could be excluded) was too low for a risk factor analysis.

Fifth, if there was an association between the oropharyngeal and subsequent anorectal-CT infections, it could reflect a higher risk of oropharyngeal CT due to risk behaviour, instead of a true association between the two types of CT infections. However, adjusting for confounding factors could have led to an overcorrection and underestimation of the main effect. Nevertheless, correction was deemed necessary, provided unique insight into the relationship between oropharyngeal and anorectal-CT infections, and corrected for high-risk behaviours for STI.

Previous studies presented results in favour of the hypothesis that oropharyngeal CT could lead to anorectal CT via the GI tract [11, 12, 15, 18, 28]. Bavoil *et al*. hypothesised that oral sex could introduce CT to the GI tract, which then could infect the rectum [13]. However, most of these studies based their findings mainly on animal studies. To our knowledge, there are no epidemiological studies that look at preceding oropharyngeal and subsequent anorectal-CT infections in humans. More studies with human data are needed, with, for example, data on the routine testing of all anatomic sites and genotyping in order to compare our study findings on the oropharyngeal–anorectal CT hypothesis.

Despite these limitations, we showed robustness of our results by doing several sensitivity analyses and taking into account some of the abovementioned limitations.

Overall, it is possible that anorectal-CT infections caused by oropharyngeal-CT infections might not have surfaced in this study. However, if no such association was found in this large-scale retrospective data that included high-risk STI clinic visitors, it is unlikely that there will be an association in the general population. The external validity of this study is low, because individuals tested multiple times at the STI clinic belong to a group at high-risk for STI. Potential associations that were missed, for example, due to spontaneous clearance and the low prevalence of oropharyngeal-CT infections, would have been small and had a limited impact on public health; in other words, it would have a minor impact on transmission at the population level and on STI care in practice.

In conclusion, this large longitudinal study did not discover any risk from preceding oropharyngeal CT for subsequent anorectal-CT infection. A possible minor association with a potential impact on a limited number of individual patients cannot be ruled out, as we used an epidemiological design rather than human experiments, but the impact of such possible association on public health is likely to be small.
